# New insights into the antimicrobial mechanisms of copper touch surfaces

**DOI:** 10.1186/1753-6561-5-S6-P39

**Published:** 2011-06-29

**Authors:** B Keevil, S Warnes

**Affiliations:** 1 School of Biological Sciences, University of Southampton, Southampton, UK

## Introduction / objectives

Survival of pathogens on touch surfaces contributes to increasing incidence and spread of antibiotic resistance and infection in hospitals. One way to address this could be to use biocidal surfaces in conjunction with improved cleaning regimes. Exposure to moist copper alloy surfaces, to simulate fomite contamination, resulted in a rapid kill of significant bacterial, viral and fungal pathogens. We now report studies on dry surfaces with a range of pathogens to elucidate the antimicrobial mechanism.

## Methods

Clinical isolates of VRE, MRSA, *E. coli* O157, *A. baumannii* and *Salmonella* were inoculated onto copper alloy and stainless steel surfaces. Survivors were assessed by culture on agar media, respiration using CTC reduction, membrane potential using Rhodamine 123 and cell membrane integrity using BacLight stain. Genomic and plasmid DNA integrity was determined using gel electrophoresis and a sensitive genomic fragmentation assay. Contribution of Cu(I) or Cu(II) ions, and superoxide or hydroxyl free radical to the antimicrobial effect was determined by the protective effect of copper chelators and reactive oxygen species quenchers.

## Results

Copper surface toxicity in enterococci and MRSA involved Cu(I) and (Cu(II) ion release and generation of superoxide, resulting in rapid collapse of membrane potential, arrested respiration and DNA breakdown. Fenton reaction generation of hydroxyl radicals was more important in Gram-negative bacteria and this was also accompanied by a compromised cell membrane.

## Conclusion

Contact surfaces containing copper could be useful to help prevent spread of viable pathogens. The rapid destruction of genomic and plasmid nucleic acid could prevent mutational resistance developing and also help reduce the spread of antibiotic resistance genes to receptive and potentially more virulent organisms, as well as genes responsible for virulence.

## Disclosure of interest

B. Keevil Grant/Research support from International Copper Association, S. Warnes Grant/Research support from International Copper Association.

